# How COVID-19 pandemic changed our management strategies for amyotrophic lateral sclerosis (ALS) patients: Egyptian study

**DOI:** 10.1186/s41983-021-00331-2

**Published:** 2021-10-04

**Authors:** Hebatallah R. Rashed

**Affiliations:** 1 Ain Shams University Hospital, Neurology department, Cairo, Egypt; 2 International Medical Center (IMC), New Heliopolis City, Egypt; 3 Faculty of Medicine, Ain Shams University, Ramsis St., Abbasseya, Cairo, Egypt

**Keywords:** Telemedicine, COVID-19, Pandemic, Amyotrophic lateral sclerosis, Egypt, ALSFRS-R

## Abstract

**Background:**

There are several studies that have discussed the efficacy of telemedicine with amyotrophic lateral sclerosis (ALS) patients; however, this approach is still preliminary in Egypt and in North Africa. The objective of the current study is to discuss current experience with telemedicine in monitoring patients in the specialized ALS clinic in Egypt. Efficacy of Revised Amyotrophic Lateral Sclerosis Functional Rating Scale (ALSFRS-R) in monitoring disease progression remotely will be discussed.

**Results:**

This is a prospective study. Forty-three ALS patients were included in this study in the period between July 1, 2020, and February 6, 2021. Fifty-three telemedicine encounters and 13 post-telemedicine office visits were available. None of the participating patients had COVID-19 infection. Eight patients showed decline in ALSFRS score. ALSFRS-R score reported during telemedicine encounters was confirmed during office visits. Three bulbar onset ALS patients had gastrostomy, and 2 bulbar onset ALS patients had Botox injection for drooling. All eight patients with declining ALSFRS-R were maintained on non-invasive ventilation (NIV) based on their symptoms.

**Conclusion:**

This is the first study discussing telemedicine in the field of ALS in Egypt and North Africa. ALSFRS-R showed feasibility and reliability in detecting disease progression remotely.

## Background

Telemedicine has become an effective alternative tool to follow up ALS patients without exposing them to higher risk of infection. There are several studies reporting the efficacy of telemedicine with ALS patients [1, 2]. During the pandemic, several studies have discussed this approach as an alternative tool to monitor ALS patients [3, 4]; however, this tool is not well developed and is still preliminary in Egypt as well as in other countries in North Africa.

In the current study, I discuss current experience in the specialized ALS clinic in Egypt with telemedicine in monitoring our registered ALS patients. I will also discuss the efficacy of Revised Amyotrophic Lateral Sclerosis Functional Rating Scale (ALSFRS-R) in monitoring disease progression remotely. This will be done by comparing scores of ALSFRS-Rs during telemedicine encounters to their pre-telemedicine and post-telemedicine office visits.

## Methods

This is a prospective study. All patients included in the study had received the diagnosis of ALS in our specialized ALS clinic [5–7], according to El Escorial revised criteria [8] during 2019 and early 2020. Participating ALS patients were followed up via telemedicine (after getting their approval) by a specialized neurologist in the period between July 1, 2020, and February 6, 2021. Telemedicine encounters were done via WhatsApp texts +/− phone calls, and all encounters were offered for free.

The following items have been evaluated with patients or their caregivers: Revised ALS Functional Rating Scale (ALSFRS-R) which has been validated in Arabic in previous study [5] to determine disease progression, intercurrent events to identify significant changes including depressive symptoms, possible signs and symptoms of COVID-19 infection, and compliance to physiotherapy. ALSFRS-R score through telemedicine encounters will be recorded, and then, it will be compared to their pre-telemedicine and post-telemedicine office visits. Post-telemedicine office visits includes mainly patients who showed decline in their ALSFRS-R during telemedicine encounters and other patients who were able to come to the office for regular follow-up.

Study has been approved by the local ethical committee at the neurology department. Patients provided informed consent.

## Results

Forty-three patients were included in this study. A total of 53 telemedicine encounters were done with patients or/and their caregivers, followed by 13 office encounters (8 patients whom have shown decline in ALSFRS-R and 5 stable patients). None of participating patients experienced symptoms or signs of COVID-19 infection.

Patients refused video calls as they do not feel comfortable or they do not have smartphones. Total length of encounters ranged from 15 to 20 min. All patients and caregivers were satisfied with this experience. Satisfaction of patients was due to several factors: the ease of interaction with their neurologist, they do not have to travel to the location of the clinic which usually add physical and financial burden, elimination of the financial burden of follow-up visits having these encounters free of charge, and the avoidance of unnecessary presence in crowds.

Demographics, clinical features, and changes in ALSFRS-R are shown in Table 1. Patients were predominantly males, and ALS type was mainly limb onset ALS. Interval between pre-telemedicine office visit and telemedicine encounter was 1–6 months in all patients. There was a change in regimen of medications in 6 patients, and the change was mainly in the dosage of antispasticity medications and dietary supplements. Antidepressants were prescribed to 8 patients who developed depressive symptoms during the pandemic.

**Table 1:** Demographics, clinical features, and changes in ALSFRS-R

Mean age (years) ± SD	49.4 ± 11.8
Limb onset	31/43 (72.1%)
Bulbar onset	12/43 (27.9%)
Mean ALSFRS-R (Pre-Televisit) ± SD	31.7 ± 6.9
Mean ALSFRS-R (Televisit) ± SD	29.9 ± 6.5

*ALSFRS-R* Revised Amyotrophic Lateral Sclerosis Functional Rating Scale

There was no statistical difference between ALSFRS-R pre and during telemedicine encounters (Table 2). Eight patients, 5 patients with bulbar onset ALS and 3 patient with limb onset ALS, showed decline in ALSFRS score by mean of 4.9 ± 5.8 (because of worsening in swallowing and respiratory functions in bulbar onset ALS patients and worsening of motor and respiratory functions in the limb onset ALS patients).

**Table 2:** Comparison between ALSFRS-R before and during telemedicine

		P-value
Mean ALSFRS-R (Pre-Televisit) ± SD	31.7 ± 6.9	0.1
Mean ALSFRS-R (Televisit) ± SD	29.9 ± 6.5	

*ALSFRS-R* Revised Amyotrophic Lateral Sclerosis Functional Rating Scale

Interval between telemedicine encounters and post-telemedicine office visits was 1–3 months. Post-telemedicine office visits included all patients who reported decline in their ALSFRS-R score in addition to 5 more stable patients. ALSFRS-R scores reported during telemedicine encounters were confirmed for all patients during office visit by clinical examination. Three bulbar ALS patients underwent gastrostomy for tube feeding; two bulbar ALS patients received Botox injection in salivary glands for drooling. All eight patients with declining ALSFRS-R were maintained on non-invasive ventilation (NIV) based on their symptoms (Table 3).

**Table 3:** Intervention for patients with declining ALSFRS-R

Bulbar ALS	62.5%
Gastrostomy	3/8 (37.5%)
Botox injection	2/8 (25%)
NIV	8/8 (100%)

*NIV* non-invasive ventilation

## Discussion

Management of patients with amyotrophic lateral sclerosis (ALS) has changed significantly during the COVID-19 pandemic. Telemedicine has become an effective tool to monitor ALS patients during the pandemic without exposing them to the risk of infection due to close contact [9]. This approach does not benefit only patients, but it also benefits healthcare professionals and decreases economic burden of the pandemic on the entire healthcare system which has to deal with the increasing number of COVID-19 patients.

The current study is the first study discussing the importance of telemedicine encounters with ALS patients in Egypt and in North Africa during the pandemic. This study included 43 patients who are already registered in the specialized ALS clinic in Egypt. These encounters were free of charge and were offered to all patients in the period between July 1, 2020, and February 6, 2021, via WhatsApp texts +/− phone calls. All patients and caregivers were satisfied with telemedicine encounters because of decreased financial burden of the disease (by eliminating the cost of transport and follow-up visits) and avoidance of unnecessary contact. Depressive symptoms were detected in 8 patients, and antidepressants were prescribed. Patients who showed decline in ALSFRS-R experienced intercurrent events as motor, bulbar, and respiratory functions which required intervention with invasive (gastrostomy and Botox injection) and non-invasive (NIV) measures. None of the patients showed symptoms suggestive of COVID-19 infection.

Participating patients and their caregivers did not feel comfortable with video calls, which also has been seen in Italy in a previous study by Capozzo and his colleagues [3], and this could be attributed to cultural reasons shared by some Mediterranean regions. Some studies have shown that the rate of depression and anxiety has increased in population [10, 11] especially among chronically ill patients [11, [12]]; in the current study, I detected depressive symptoms in some of the ALS patients, which has been also found in some Italian studies [4, 13].

One of the limitation of this study is being unable to re-assess all patients face to face with ALSFRS-R after telemedicine encounters to validate their ALSFRS-R scale results; only patients who reported decline in ALSFRS-R sale were brought to the office and re-assessed, which limits the detection of worsening of symptoms. However, ALSFRS-R was sensitive in detecting disease progression which mandated intervention.

The importance of this study comes from being the first study showing the efficacy of telemedicine in the field of ALS in Egypt and North Africa, which was not well developed before the pandemic. ALSFRS-R which was validated in Arabic previously [5] showed feasibility and was easy to administer to patients and their caregivers in telemedicine encounters, and helped in detecting disease progression which mandated intervention.

## Conclusion

In conclusion, this study supports other studies [3, 4], and its preliminary result shows that telemedicine is an effective and easy tool to monitor ALS patients who cannot come to office for regular visits. I recommend that telemedicine using ALSFRS-R scale should be integrated in the standard of care for ALS patients especially for those with physical disability and cannot be transferred to hospitals for follow-up visits. Larger study is still needed to compare post-telemedicine in-office ALSFRS-R scale results to telemedicine results.

## Data Availability

The data that support the findings of this study are available from the corresponding author upon reasonable request.

## Abbreviations

ALS: Amyotrophic lateral sclerosis
ALSFRS-R: Revised Amyotrophic Lateral Sclerosis Functional Rating Scale
NIV: Non-invasive ventilation
